# Electrophysiological Responses to Expectancy Violations in Semantic and Gambling Tasks: A Comparison of Different EEG Reference Approaches

**DOI:** 10.3389/fnins.2018.00169

**Published:** 2018-03-20

**Authors:** Ya Li, Yongchun Wang, Baoqiang Zhang, Yonghui Wang, Xiaolin Zhou

**Affiliations:** ^1^School of Psychology, Shaanxi Normal University, Xi'an, China; ^2^School of Psychological and Cognitive Sciences, Peking University, Beijing, China; ^3^Beijing Key Laboratory of Behavior and Mental Health, Peking University, Beijing, China; ^4^Key Laboratory of Machine Perception (Ministry of Education), Peking University, Beijing, China; ^5^PKU-IDG/McGovern Institute for Brain Research, Peking University, Beijing, China

**Keywords:** expectancy violation, reference electrode standardization technique (REST), average reference (AVE), linked mastoid (LM), N400, feedback-related negativity (FRN)

## Abstract

Dynamically evaluating the outcomes of our actions and thoughts is a fundamental cognitive ability. Given its excellent temporal resolution, the event-related potential (ERP) technology has been used to address this issue. The feedback-related negativity (FRN) component of ERPs has been studied intensively with the averaged linked mastoid reference method (LM). However, it is unknown whether FRN can be induced by an expectancy violation in an antonym relations context and whether LM is the most suitable reference approach. To address these issues, the current research directly compared the ERP components induced by expectancy violations in antonym expectation and gambling tasks with a within-subjects design and investigated the effect of the reference approach on the experimental effects. Specifically, we systematically compared the influence of the LM, reference electrode standardization technique (REST) and average reference (AVE) approaches on the amplitude, scalp distribution and magnitude of ERP effects as a function of expectancy violation type. The expectancy deviation in the antonym expectation task elicited an N400 effect that differed from the FRN effect induced in the gambling task; this difference was confirmed by all the three reference methods. Both the amplitudes of the ERP effects (N400 and FRN) and the magnitude as the expectancy violation increased were greater under the LM approach than those under the REST approach, followed by those under the AVE approach. Based on the statistical results, the electrode sites that showed the N400 and FRN effects critically depended on the reference method, and the results of the REST analysis were consistent with previous ERP studies. Combined with evidence from simulation studies, we suggest that REST is an optional reference method to be used in future ERP data analysis.

## Introduction

To perform efficiently in a changing world, we must rapidly evaluate the outcomes of our actions or thoughts to guide future behaviors or thoughts. According to previous event-related potential (ERP) studies, feedback-related negativity (FRN) is the most important component associated with outcome evaluations. FRN is a negative-going component that is usually the largest over a fronto-central electrode site between 250 and 300 ms after the onset of a feedback stimulus (Miltner et al., 1997; Gehring and Willoughby, 2002; Holroyd and Coles, 2002; Nieuwenhuis et al., 2004; Yeung et al., 2005; Heldmann et al., 2008). FRN is typically observed in response to unexpected outcomes, such as money losses (Gehring and Willoughby, 2002), incorrect responses (Miltner et al., 1997) and unexpected negative feedback (Nieuwenhuis et al., 2004). Moreover, a previous study showed that the unexpected perceptual incompatibility between an expected color and the color that was actually presented could also elicit FRN (Jia et al., 2007). The expectancy-deviation hypothesis proposes that FRN is elicited by a mismatch between the expected feedback and the actual feedback (Oliveira et al., 2007). However, consensus regarding the role of the FRN component in outcome evaluations is lacking. It is still unknown whether FRN codes a violation of expectancies in general.

In a previous study, Roehm et al. (2007) investigated the ERP responses to the expectancy violation in an antonym relation task. Specifically, antonym word pairs were presented in the fixed context as “*The opposite of X is Y*.” The participants were asked to indicate the correctness of the sentence. In this task, the second word (Y) of the antonym word pair served as the feedback to the prediction of the first word (X). The authors found that an expectancy deviation under an antonym relations context could induce a negative component with a peak at approximately 300 ms. Although the authors considered this negative component N400, the negative component in this study occurred earlier and had a more anterior distribution than the typical N400 component. The profile of this component was similar to that of the FRN component. In addition, FRN is likely elicited by expectancy deviations under the antonym relations context since an antonym relations violation is also a type of expectancy deviation. However, the profile of the so-called “N400” in the antonym study was obtained under conditions that differ from those used in FRN studies. For example, the EEG recording method, the analysis method and the participants all differed across these studies. We could not directly compare the components observed in the different research studies. Hence, additional studies using the same methods and a within-subjects experimental design are required to address this issue. These studies could extend our understanding of the nature of the FRN component.

Furthermore, the choice of reference approach could influence the observed EEG data. The “choice of EEG reference point” has been an issue for many years. The EEG reference method choice is crucial since the potential measured at each electrode on the scalp is calculated as the potential difference between the active electrode site and the reference site. This method assumes that the recorded data at the reference site cannot be contaminated by brain activity. However, this approach cannot be guaranteed when the reference site is on the scalp. Previous FRN and N400 studies have mainly utilized the linked mastoid (LM) as a reference (Gehring and Willoughby, 2002; Roehm et al., 2007), which is calculated as the average of the signals from the left and right mastoids. However, previous simulation studies have shown that the scalp distribution of LM-obtained EEG data has significant distortions (Yao, 2001; Yao et al., 2005; Qin et al., 2010) because the EEG signal of the left and right mastoids is influenced by physiologically dynamic signals from the brain. In addition, no point on the scalp or body surface always has a zero potential. Hence, the choice of the EEG reference method might influence the spatial and temporal profiles of the observed N400 and FRN components.

Fortunately, accumulating evidence suggests that the reference electrode standardization technique (REST) should be used as a neutral reference-free approach in EEG studies (Yao, 2001; Tian and Yao, 2013). The premise behind the REST approach is to re-reference the EEG or ERP data to a neutral infinite reference point that is unlikely to be influenced by brain activity through a transfer matrix (Yao, 2001; Yao et al., 2005). The transfer matrix is computed by using a non-unique equivalent dipole source model and a proposed three-concentric-sphere head model. Given these merits, the REST approach was used in this study to avoid the possible influences of the reference method and to confirm the observed results. Moreover, the AVE reference approach, which is calculated as the average of all electrode sites, is also commonly used as a reference-free approach in EEG studies. However, the influence of the AVE reference procedure on the N400 and FRN effects is still unknown.

To determine whether the expectancy violation under an antonym relations context could induce FRN, the current study directly compared the components elicited by expectancy violations in an antonym relations task and gambling task using a within-subjects experimental design. Moreover, the current study examined the influence of the choice of ERP reference approach on the amplitude and scalp distribution of the experimental effects. Hence, the current study compared the effects of the LM, REST, and AVE reference approaches on the amplitude and scalp distribution of the ERP effects as the expectancy violation increased.

## Methods

### Participants

Twenty-three naïve participants (11 males, 12 females; mean age = 22; *SD* = 2.9) with normal or corrected-to-normal vision were recruited for this experiment. All participants were right-handed without any known neurological or visual disorders. Each participant received a basic payment of 50 yuan for his/her participation, and the participants were informed that they would receive an additional bonus based on their performance in the gambling task. The participants provided written informed consent before the experiment. The study was approved by the local research ethics committee.

### Stimuli and procedure

#### Gambling task

##### Procedure

The stimuli were displayed on a black uniform background on a 22-inch CRT monitor with a refresh rate of 60 Hz and a resolution of 1,024 × 768 pixels. The stimuli and procedures were programmed using Presentation (Neurobehavioral System Inc., San Francisco, CA). The participants viewed the stimuli at a distance of 100 cm in a dimly lit room, and their heads were stabilized by a chin-and-head rest.

**Figure 1:**
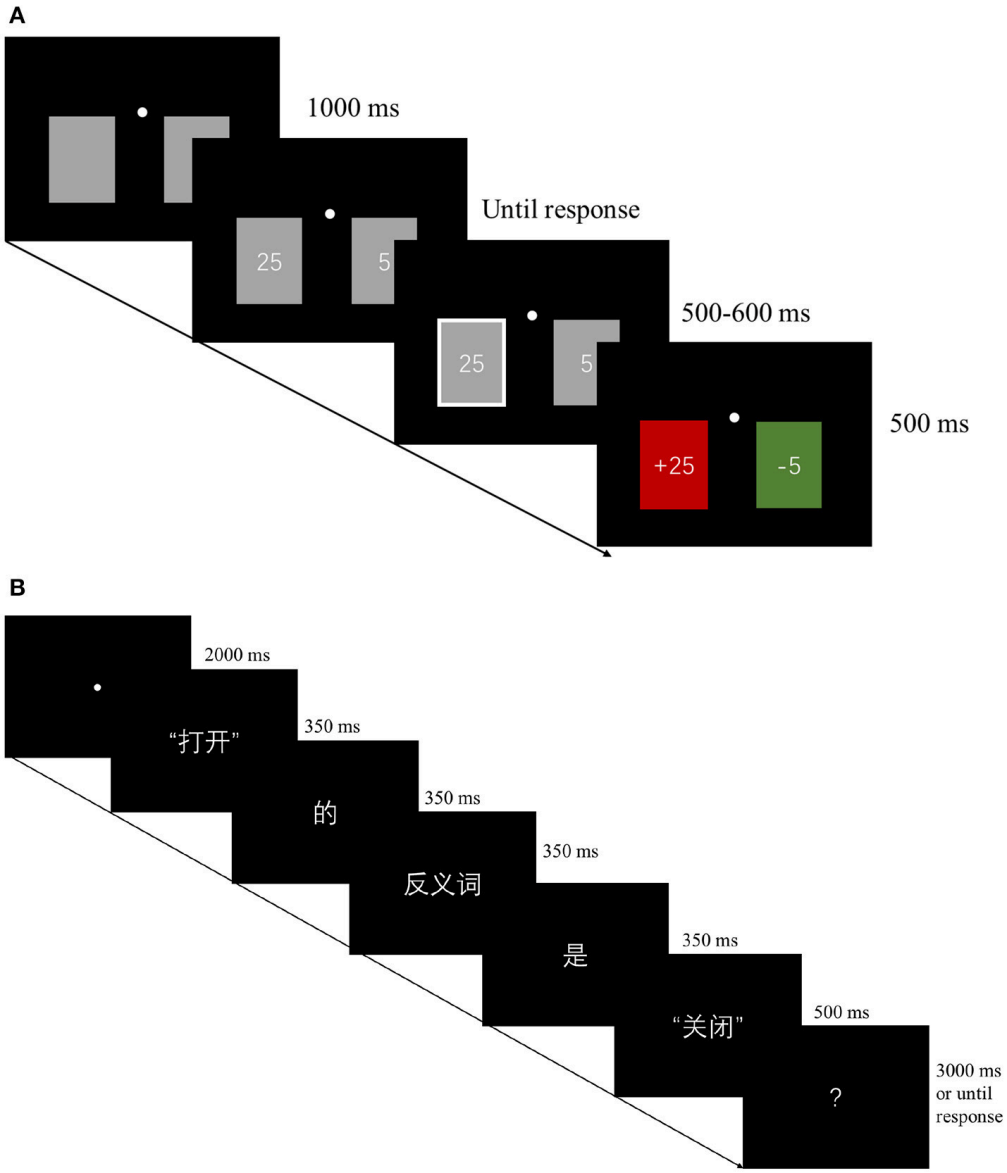
Experimental procedures. **(A)** An example trial of the gambling task. The four possible number combinations (i.e., 25/25, 5/5, 5/25, and 25/5) was shown at the center of the gray cards until response. The inter-trial interval was 1,000 ms. **(B)** An example trial of the antonym task. The illustration shows “The opposite of *open* is *close*” in Chinese (word-by-word translation: “打开*(open)*/的(of)/反义词(opposite)/是(is)/关闭*(close)*”). The ISI between each word was 200 ms, except that the ISI between the word *is* and the critical stimuli (Y) was 200–300 ms with a 50-ms per jitter step. After a 750 ms ISI (range: 500–1,000 ms), a question mark (?) was shown until a response was given or 3 s had elapsed. The inter-trial interval was 2,250 ms.

As shown in Figure 1A, each trial started with the presentation of two gray cards on a uniform black background (2.3° wide, 3.2° high) on the left and right sides of a fixation dot (3.6° away from the fixation dot) for 1,000 ms. Then, the number 5 and/or the number 25 was shown at the center of the gray cards. Four possible combinations (i.e., 25/25, 5/5, 5/25, and 25/5) were presented at an equal probability. The numbers remained until a response was given. After the response, the white outline of the card thickened for 550 ms (range: 500–600 ms) to confirm the selection. Finally, the chosen card became red or green to inform the participant of the outcome of the current trial. To emphasize the valence of the outcome, the “+” or “–“ symbol was added before the number to indicate the gain or loss, respectively. The inter-trial interval was 1000 ms. The participants were informed that they could be rewarded or penalized the amount of money indicated by the feedback. In addition, the cumulative outcome across each trial was presented at the end of the experiment. The experiment consisted of two blocks of 160 trials each. A practice block was performed before the formal test.

Unknown to the participants, the sequence of different types of trials was pseudorandomized, and no more than 4 consecutive trials were from the same condition. The gain and loss status of the participants' chosen cards was predetermined before the experiment at an equal probability. The assignment of the green/red colors to represent the gain/loss was counterbalanced between participants.

### Antonym task

#### Materials

One hundred and twenty sets of the three experimental conditions (i.e., antonyms, related and unrelated) were used in the main experiment, resulting in a total of 360 critical stimuli. The word frequency and strokes of the critical stimuli are shown in Table 1. Repeated-measures analyses of variance (ANOVAs) revealed that the differences among the three conditions were not significant [Frequency: *F*_(2, 357)_ = 0.31, *p* > 0.74; Stroke: *F*_(2, 357)_ = 1.62, *p* > 0.20]. To ensure that no single word was repeated for any particular participant, all critical stimuli were divided into three lists, and each participant was presented with stimuli on one particular list. To balance the responses, 40 additional antonym pairs serving as filter conditions were added to each list. Thus, each list contained 160 items (i.e., 80 antonym pairs, 40 related pairs and 40 unrelated pairs). The assignment of each list was counterbalanced across participants using a Latin square design.

**Table 1:** Mean rating scores in both groups.

**Condition**	**Stroke**	**Frequency**	**Antonymy**	**Relatedness**
	**Mean (SD)**	**Mean (SD)**	**Mean (SD)**	**Mean (SD)**
A. Antonyms	17.65 (5.47)	32.18 (43.76)	1.32 (0.26)	1.48 (0.25)
B. Related	17.42 (4.24)	28.71 (51.29)	5.16 (1.18)	2.84 (0.79)
C. Unrelated	16.63 (4.64)	27.92 (34.01)	6.55 (0.25)	6.07 (0.50)

#### Questionnaire pretest

To determine whether the experiment conditions indeed differed from each other in terms of antonymy and semantic relatedness, we conducted a questionnaire pretest. Seventy-two participants (28 males, 44 females) completed the questionnaire. The participants were randomly and averagely assigned to two groups (Group A: 23 females, age: 22 ± 2.4; 13 males, age: 23 ± 2.2. Group B: 21 females, age: 22 ± 2.2; 13 males, age: 23 ± 2.6.). No significant age difference was observed between Groups A and B, F (1.36) = 0.003, *p* > 0.96.

Both groups were instructed to rate the relationship of each word pair on a 7-point scale. The only difference between these two groups was the judgment task. Group A was tasked with judging *the degree of antonymy* (i.e., the degree to which the pair could be considered an antonym pair, 1: optimal antonymy, 7: not at all an antonym), while Group B was tasked with judging *the degree of relatedness* between the two words (1: very strongly related, 7: completely unrelated). Other than the judgment task, the questionnaires were identical in both groups. Both groups completed three versions of the questionnaire including 160 word pairs (antonym pairs: 80; related word pairs: 40; and unrelated word pairs: 40). The assignment of each version was counterbalanced across the participants using a Latin square design. No single word was repeated in each version. Thus, 12 participants rated the degree of relatedness and antonymy of each word pair.

The rating scores of both groups are shown in Table 1. Repeated-measures ANOVAs with two factors (experiment condition: antonym, related and unrelated; group: antonymy judgment, relatedness judgment) revealed a main effect of group, *F*_1(1, 22)_ = 100.78, *p* < 0.001, *F*_2(1, 238)_ = 351.56, *p* < 0.001, a main effect of condition, *F*_1(1, 22)_ = 858.77, *p* < 0.001, *F*_2(1, 238)_ = 1,433.85, *p* < 0.001, and a significant interaction between condition and group, *F*_1(1, 22)_ = 194.73, *p* < 0.001, *F*_2(1, 238)_ = 324.64, *p* < 0.001. Pairwise comparisons revealed that the differences between any two conditions (i.e., antonym vs. related, antonym vs. unrelated, and related vs. unrelated) were all significant (all *p* < 0.001) in both groups. In summary, these results confirmed that the stimuli sets of the three conditions differed along the two desired dimensions of *antonymy* and *relatedness*.

#### Procedure

As shown in Figure 1B, each trial started with a 2,000-ms fixation period, followed by a sentence that was presented word by word in the fixed sequence *The opposite of X is Y* in Chinese. Each word, except for the word Y, was presented for 350 ms, while the word Y was shown for 500 ms to reduce the off-stimulus influence on the ERP signal. The ISI between each word, except for the ISI between the word *is* and the critical stimuli (Y), was 200 ms. The mean duration of the ISI between the word *is* and the critical stimuli (Y) was 250 ms with a 50-ms per jitter step (range: 200–300 ms). After a 750 ms ISI (range: 500–1,000 ms) with a black background only, a question mark (?) was shown until a response was given or 3 s had elapsed. The inter-trial interval was 2,250 ms. The participants were asked to indicate whether the sentence was correct or incorrect as quickly and accuracy as possible.

Unknown to the participants, the sequence of the different types of trials were pseudorandomized, no more than 3 consecutive trials represented the same condition and no more than 5 trials had the same responses of “yes” or “no.” The experiment consisted of two blocks of 80 trials each. A practice block of 32 trials was performed before the formal test.

### Electrophysiological recordings

The electroencephalogram (EEG) data were recorded using a 64-channel EEG cap according to the international 10/20 system (Brain Products, Munich, Germany), and the reference electrode was placed on the tip of the nose. The horizontal and vertical electrooculograms (EOG) were monitored via electrodes placed lateral to the external canthus of the left eye and above the right eye, respectively. The AFz electrode on the cap served as a ground. During the recordings, all electrode site impedances were maintained below 10 kΩ. The EEG data were recorded at a sampling rate of 500 Hz and amplified using a 0.016–100 Hz bandpass for offline analysis.

### Data analysis

The offline data preprocessing was performed using Brain Vision Analyzer 2.0 (Brain Products, Munich, Germany). The EEG data were re-referenced using the average mastoids. The ocular artifacts were corrected using an eye-movement correction algorithm (Gratton et al., 1983). Then, the continuous EEG data were separated into epochs from −200 to 800 ms around the onset of the feedback stimuli. The baseline was defined as the epoch from 200 to 0 ms before the stimulus onset. Baseline corrections were performed by subtracting the average activity of each channel during the baseline period from each sample. All epochs in which the EEG voltages exceeded the threshold of ±70 μV were excluded from further analysis. The EEG data were low-pass filtered below 30 Hz (decay of stop band: 24 dB per octave). The remaining epochs with correct responses were then averaged across trials according to each condition. After all preprocessing steps were completed, the ERP data were re-referenced using the AVE (computed as the average of all channels) and REST methods. This step was performed to effectively maintain the same standard of artifact removal across all reference approaches.

All further analyses were performed using EEGLAB, ERPLAB and/or the REST toolbox (Yao, 2001; Dong et al., 2017; Hu et al., 2018) in a MATLAB environment. For the gambling task, the ERP experimental effect was calculated by subtracting the gain condition (expected condition) from the loss condition (unexpected condition). For the antonym relations task, the experimental effect of expectancy violation was measured by subtracting the antonym condition (expected condition) from the related and unrelated conditions (unexpected condition). All further statistical analyses were performed based on these difference waves.

The statistical analyses were conducted using both mass univariate analysis and multifactor statistical analyses. To assess the component induced in the antonym relations task and gambling task in an unbiased manner, a mass univariate approach was performed to determine exactly when and where the ERP effect appeared (Groppe et al., 2011; Luck and Gaspelin, 2017). Specifically, one-sample *t*-tests (compared with zero) were conducted for each difference wave (deviation condition–baseline condition) at each time bin and electrode site. The permutation-based strong control of the familywise error rate (FWER) was performed to correct for the multiple comparisons. Thus, the ERP effect was confirmed based on the time window and electrode sites that showed a significant effect.

To determine the influence of the reference method on the experimental effect across sites, the identified ERP effects were entered into a repeated-measures ANOVAs with experimental condition, reference method (LM, REST, and AVE) and topographical factors (Fz, FCz, Cz, CPz, and Pz) entered separately for the gambling task and the antonym relations task. The midline electrodes were chosen because the FRN effect and the N400 effect have been shown to be the greatest at these electrodes in previous studies. The Greenhouse-Geisser adjustment for non-sphericity was applied as appropriate. *Post hoc* tests and one-sample *t*-tests were also conducted, and *p*-values were corrected by performing Bonferroni adjustments to avoid multiple comparisons errors.

## Results

### Behavioral results

The mean accuracy is shown in Table 2. Regarding the accuracy, a repeated-measures ANOVA revealed a main effect of experimental condition, *F*_(2, 46)_ = 55.70, *p* < 0.001, partial η^2^ = 0.71. Pairwise comparisons showed that the performance under the antonyms and unrelated conditions was better than that under the related condition (antonyms vs. related: 0.12, *SE* = 0.02, *p* < 0.001; unrelated vs. related: 0.14, *SE* = 0.02, *p* < 0.001). Regarding the reaction time (RT), a repeated-measures ANOVA revealed a main effect of experimental condition, *F*_(2, 46)_ = 13.6, *p* < 0.001, partial η^2^ = 0.37. Pairwise comparisons showed that the performance under the antonyms and unrelated conditions was better than that under the related condition (antonyms vs. related: 59 ms, *SE* = 16 ms, *p* < 0.01; unrelated vs. related: 88 ms, *SE* = 22 ms, *p* = 0.001). These behavioral results showed higher accuracy and faster reaction times for the antonyms and unrelated conditions than for the related condition.

**Table 2:** Behavioral results for the antonym task.

**Condition**	**Accuracy**	**RT (ms)**
	**Mean (SD)**	**Mean (SD)**
A. Antonyms	0.98 (0.29)	441 (165)
B. Related	0.86 (0.77)	498 (204)
C. Unrelated	0.99 (0.15)	412 (165)

*RT, Reaction time*.

### Mass univariate analysis

**Figure 2:**
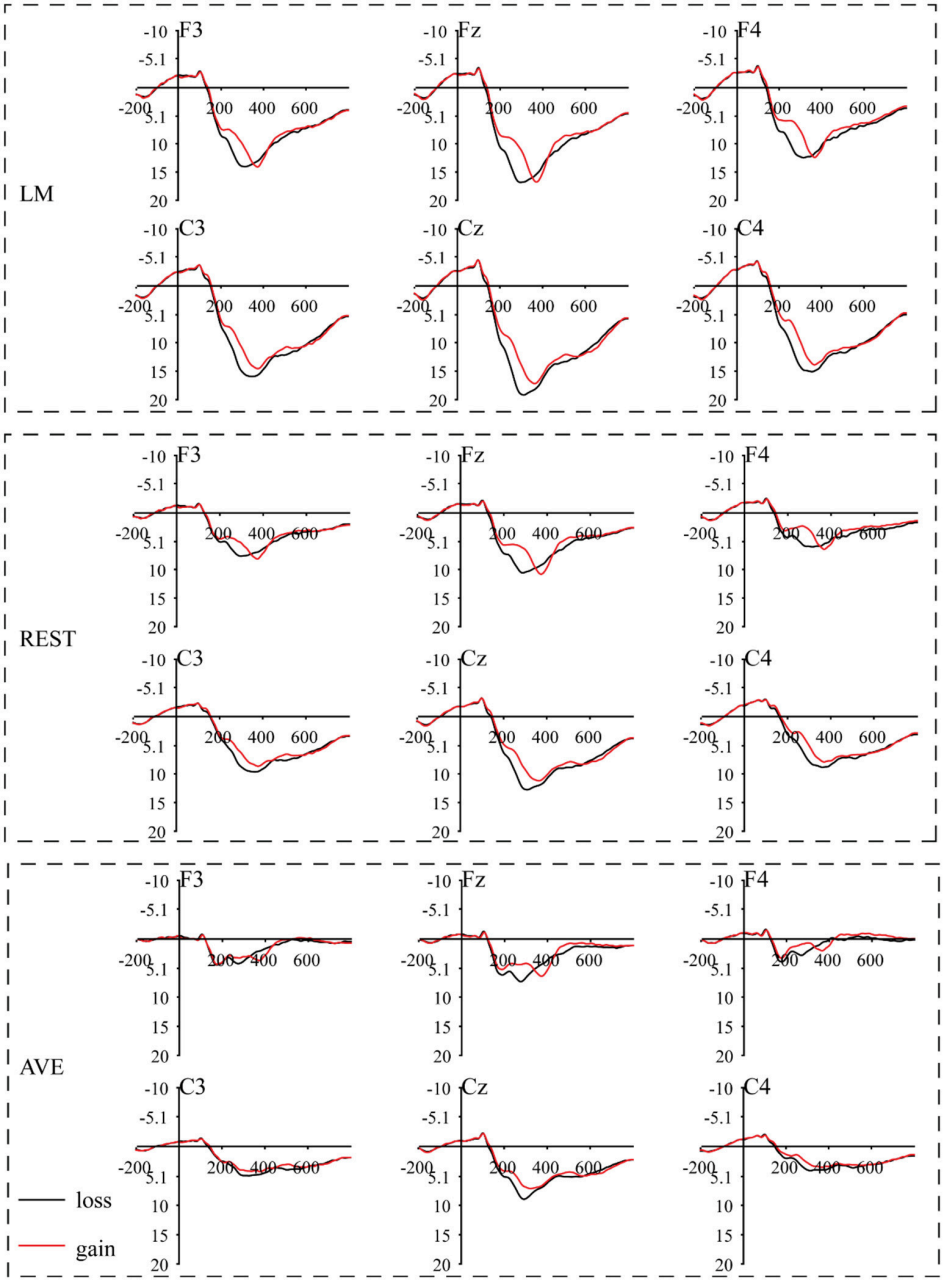
Grand-average event-related potential (ERP) waveforms under the gain and loss conditions in a gambling task using three separate reference approaches. The feedback stimulus onset occurred at 0 ms. Negativity is plotted upward.

**Figure 3:**
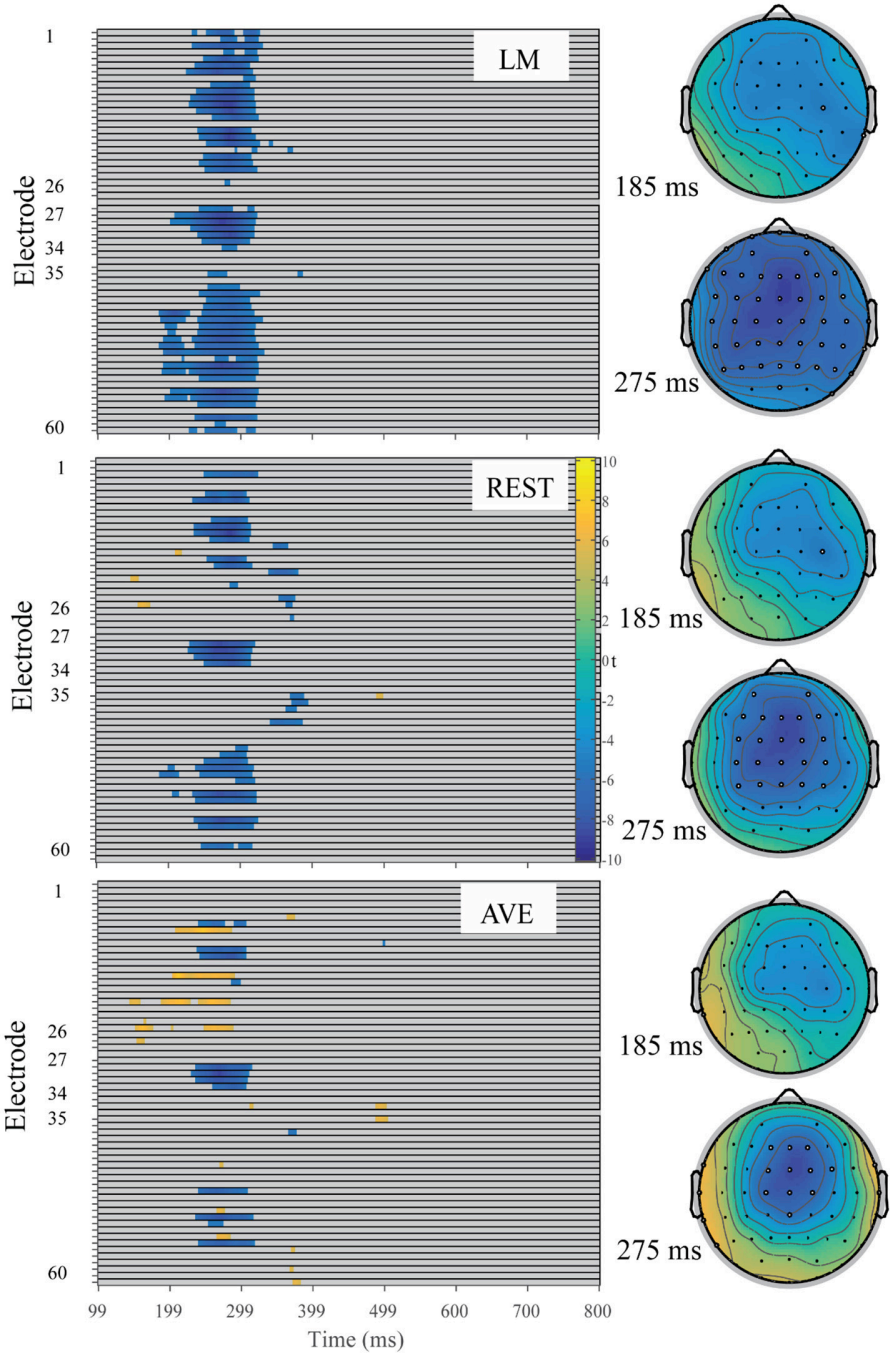
Raster diagram illustrating the significant FRN effects according to *t*-test permutation tests using three reference approaches. Blue and yellow rectangles indicate the time points and electrode sites at which the ERP effects are significantly smaller and larger than zero, respectively. Gray rectangles indicate time points and electrode sites at which no significant differences were found. Right: the corresponding *t*-value topographical maps at 185 and 275 ms for each reference approach. The white electrode indicates the significant FRN effect at that site while the black electrode indicates no significant at that moment.

A mass univariate analysis was first conducted to analyze the differences in the waveforms in each task with the LM reference method to maintain the same reference method used in previous studies (Gehring and Willoughby, 2002; Roehm et al., 2007). Specifically, we performed *t*-tests at each time point and each electrode site to analyze the different waveforms to assess the distribution of the experimental effects over the electrode sites. As shown in Figure 2, a more negative component was observed under the loss condition compared with that observed under the gain condition in the gambling task. The mass univariate analysis showed that the significant difference was mainly focused at 180-330 ms and peaked at 275 ms (Figure 3). The experimental effect was the largest at the fronto-central recording sites. The early negative component observed in our study is consistent with the FRN effect defined by previous studies (e.g., Gehring and Willoughby, 2002; Holroyd and Coles, 2002; Heldmann et al., 2008). The observed pattern was confirmed using the REST and AVE reference methods. Thus, the current study replicated the FRN effect induced by a gambling task.

**Figure 4:**
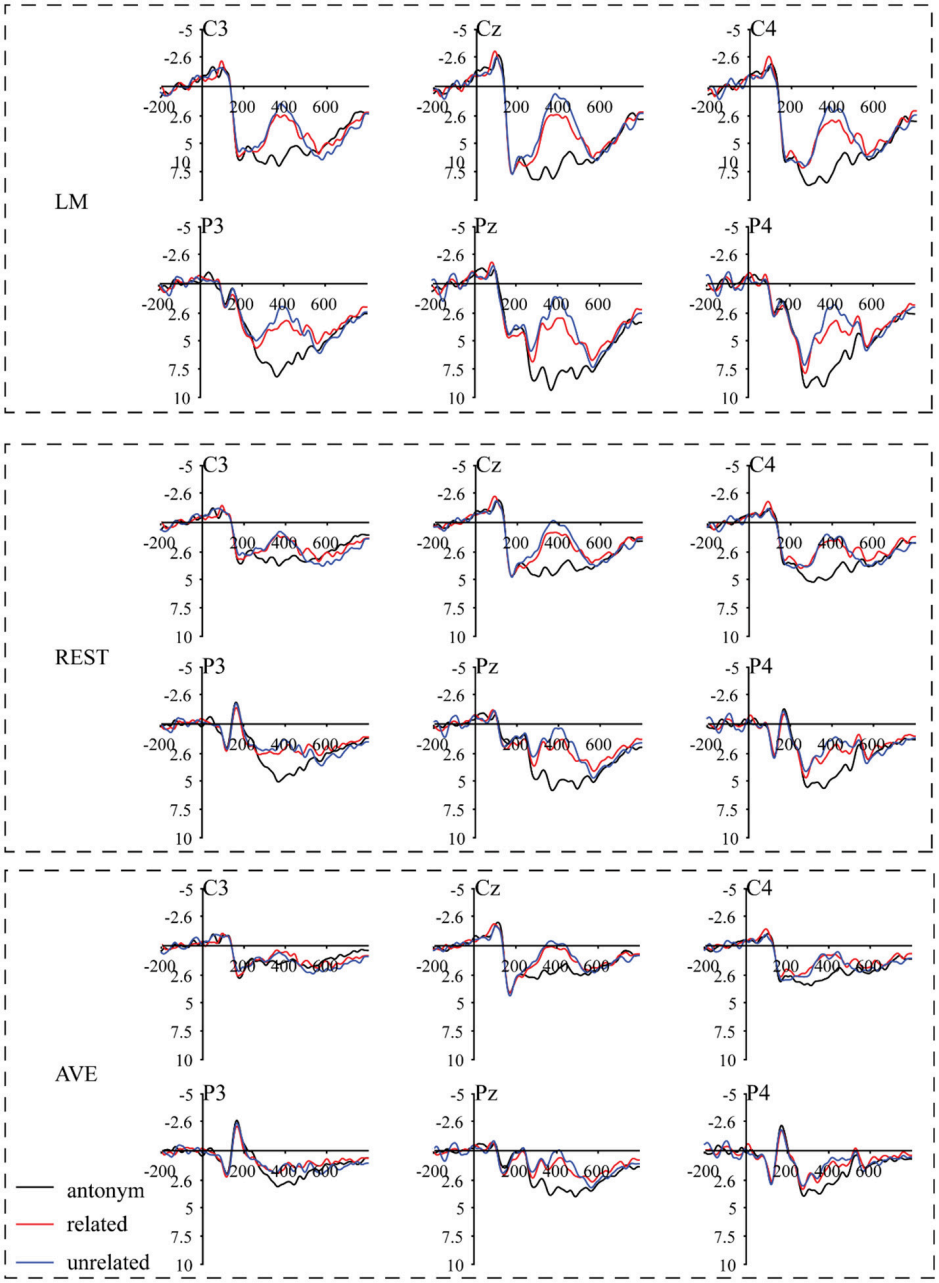
Grand-average ERP waveforms under the antonyms, related and unrelated conditions in an antonym relations task using three separate reference approaches. The second word (Y), as the feedback stimulus onset, occurred at 0 ms. Negativity is plotted upward.

**Figure 5:**
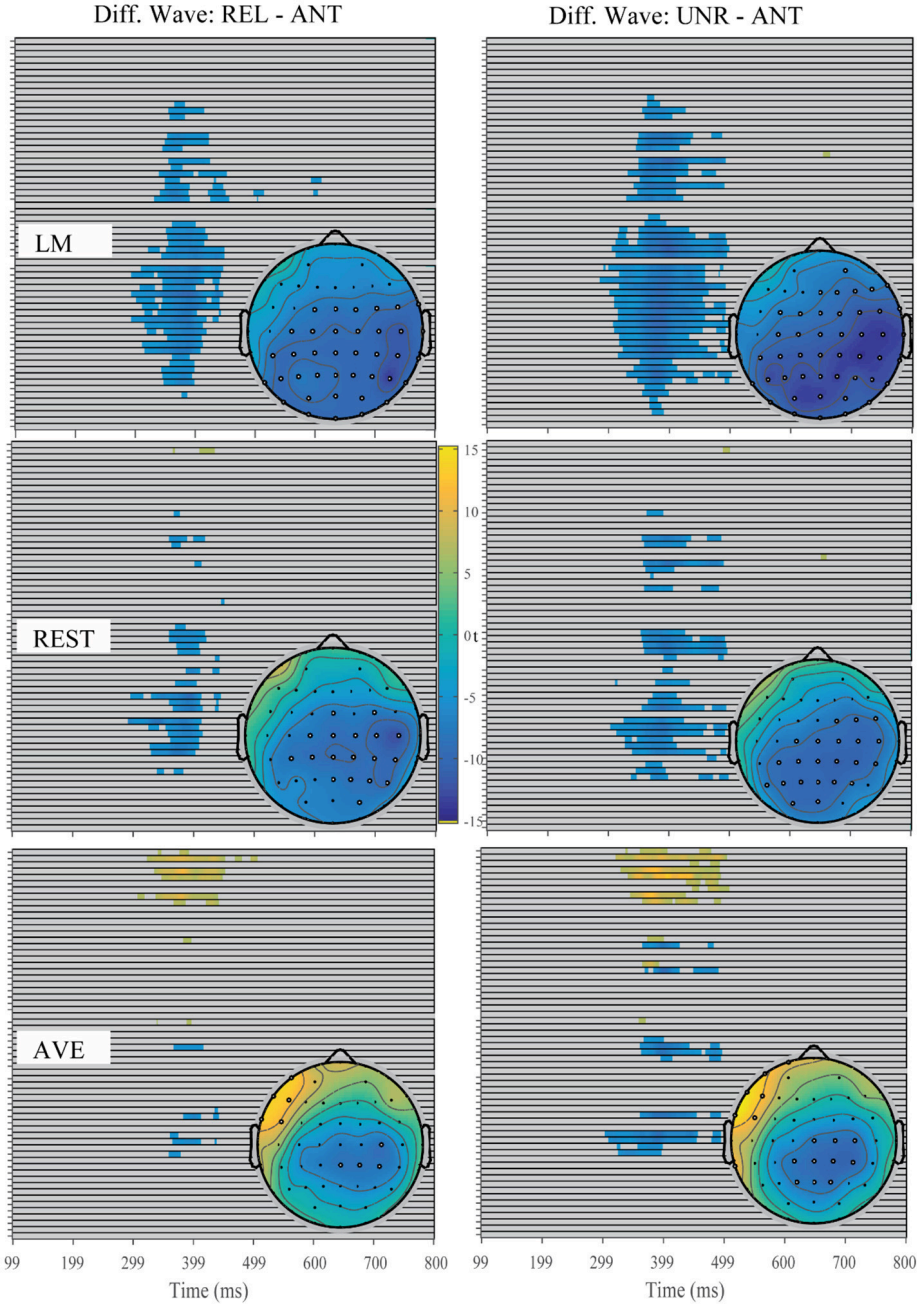
Raster diagram illustrating the significant N400 effects according to the *t*-test permutation tests using three reference approaches. Blue and yellow rectangles indicate the time points and electrode sites at which the ERP effects are significantly smaller and larger than zero, respectively. Gray rectangles indicate the time points and electrode sites at which no significant differences were found. The inset topographical maps reveals the corresponding *t*-value maps at 370 ms for each experimental condition and each reference approach. The white electrode indicates the significant N400 effect at that site while the black electrode indicates no significance at that moment.

Figure 4 shows that the waveform under the unrelated and related conditions was more negative-going than that under the antonym condition in the antonym task. The mass univariate analysis showed that the significant difference was focused at 290–500 ms and peaked at 370 ms (Figure 5). The negative component observed in our study was consistent with the N400 effect defined by previous studies (e.g., Kutas and Hillyard, 1980; Luck, 2014) and was confirmed using the REST and AVE reference methods. Thus, the negative component induced by the antonym task was an N400 effect rather than an FRN effect, and the N400 effect indicates an unexpected sematic prediction violation process.

For the FRN effect, the electrode sites of the revealed significant effects were different among all three reference approaches. Specifically, the significant electrode sites were widely distributed using the LM reference approach and focused in the front-central electrode sites using the REST reference approach. In addition, the AVE-obtained FRN effect was reversed in polarity (positive amplitude) at certain electrode sites. For the N400 effect, the electrode sites showing significant effects were also widely distributed in the LM reference method, while the significant effects were mainly focused in the posterior electrode sites using the REST reference approach. However, the AVE-obtained N400 effect was reversed in polarity at the right frontal electrode sites. These results suggested that the sites of the revealed significant effects were dependent on the choice of reference approach.

In summary, the time window and site of the revealed negative components suggest that the ERP effects induced by the prediction violations differed between the gambling task and the antonym task. The observed patterns were confirmed by using all three reference methods, suggesting that the ERP components induced by these two tasks differ.

### Multifactor statistical results

#### FRN effect and N400 effect

**Figure 6:**
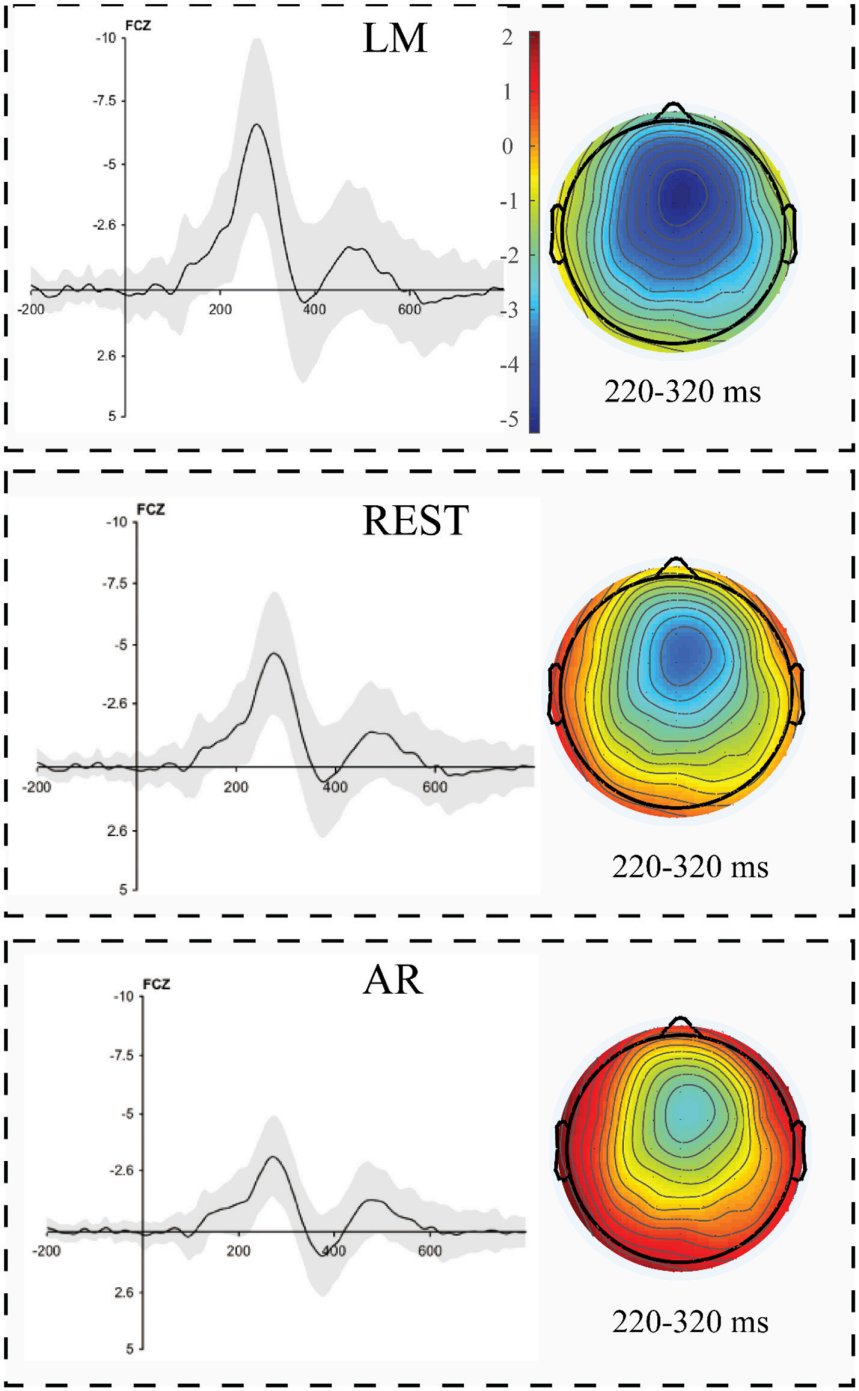
The loss minus gain difference waveforms in a gambling task using three separate reference approaches. The corresponding topographical maps of the FRN effect (180–330 ms) revealed a strong fronto-central distribution using all three reference approaches. The shading indicates the standard error across the participants.

A visual inspection of the data showed an FRN effect under the gambling task using all three reference methods. Based on visual inspection and results from the mass univariate analysis, the FRN effects were calculated as the mean amplitude of the loss-minus-gain difference wave between 220 and 320 ms (Figure 6). One-sample *t*-tests were performed to statistically examine the reliability of the FRN effect at each electrode site across all reference methods in both tasks. In the gambling task, using the LM reference method, the FRN effect was significant at all electrode sites except for Oz (all other Bonferroni-corrected *p* < 0.05, Cohen's *d* > −0.78). Using the REST reference method, a significant FRN effect was observed at all electrode sites, except for POz and Oz (all other Bonferroni-corrected *p* < 0.01, Cohen's *d* > −0.96). Using the AVE reference method, a significant FRN effect was observed at the fronto-central electrode sites (i.e., Fz, FCz, Cz, and CPz, all Bonferroni-corrected *p* < 0.001, Cohen's *d* > −1.25); however, a significant positive component was observed at Oz (Bonferroni-corrected *p* < 0.05, Cohen's *d* > 0.93). In contrast, in the antonym task, the FRN effect was not significant at all electrode sites across all reference methods (smallest Bonferroni-corrected *p* = 0.06; all other ps > 0.2). These results suggested that the unexpected outcomes induced the FRN effect in the gambling task and that this effect was confirmed by all reference approaches.

**Figure 7:**
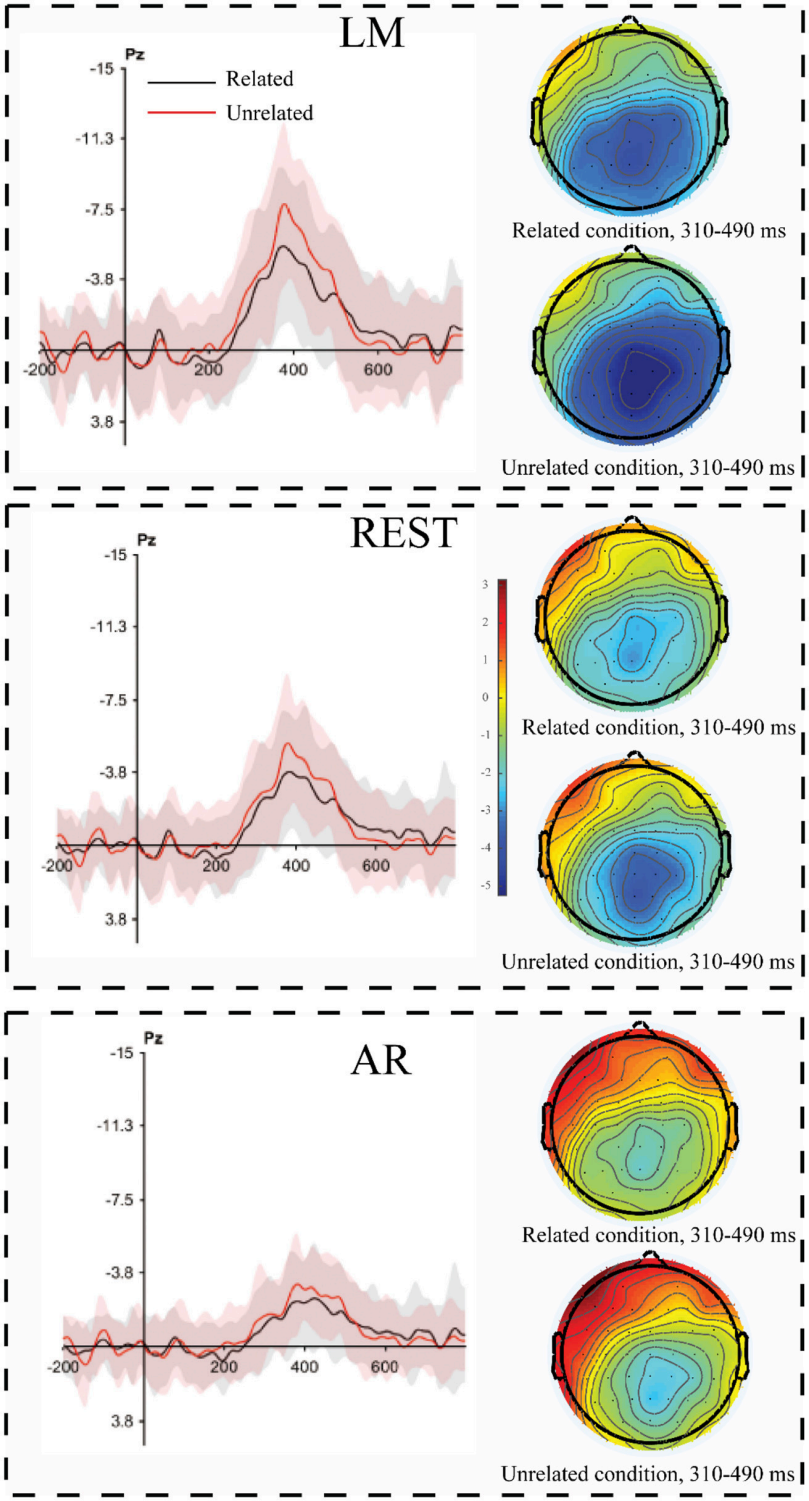
The violation minus antonym difference waveforms as a function of the magnitude of the expectancy violation. The corresponding topographical maps of the N400 effect (310–490 ms) for large violations (unrelated condition) and small violations (related condition) revealed a strong central-parietal distribution across all three reference approaches. The shading indicates the standard error across the participants.

A visual inspection of the data showed a typical N400 effect under the related and unrelated conditions using both the LM and REST reference methods, while the AVE method revealed the opposite pattern, with a negative amplitude at the posterior electrode sites and positive amplitudes at the frontal electrode sites (Figure 7). One-sample *t*-tests were conducted to statistically examine whether the N400 effect was induced under each experimental condition at each electrode site using all reference methods in both tasks. According to the one-sample *t*-tests with Bonferroni corrections, the mean amplitude of the N400 effect (310–490 ms) under the related condition was significant at all sites, except for Fz, using the LM and REST reference methods (LM: all other Bonferroni-corrected ps < 0.008, Cohen's *d* > −0.97; REST: all other Bonferroni-corrected ps < 0.05, Cohen's *d* > −0.82), and the N400 effect obtained with the AVE method was significant at Cz, CPz, Pz, and POz (all Bonferroni-corrected ps < 0.05, Cohen's *d* > −0.80). Under the unrelated condition, the N400 effect was significant at most sites using all three reference methods (LM: all except for Fz, all other Bonferroni-corrected ps < 0.008, Cohen's *d* > −0.96; REST: all except for Fz and FCz, all other Bonferroni-corrected ps < 0.002, Cohen's *d* > −1.09; AVE: all except for Fz, FCz, and Oz, all other Bonferroni-corrected ps < 0.002, Cohen's *d* > −1.08). In the gambling task, however, it was not significant at all electrode sites across all reference methods (smallest Bonferroni-corrected *p* > 0.7). Thus, the typical N400 effect could be reliably observed under both the related and unrelated conditions in the antonym task, which was confirmed using both the LM and REST reference method. However, the AVE method could distort the experimental effect, which, in turn, could alter the results.

#### Reference effects on the amplitude and scalp distribution

To further statistically examine the influence of the reference method on the amplitude and scalp distribution of the FRN effect, we included the amplitude of the FRN effect in a two-way (electrode site × reference) repeated-measures ANOVA. The ANOVA revealed a main effect of reference method [*F*_(2, 44)_ = 306.56, *p* < 0.001, partial η^2^ = 0.75], and the pairwise comparisons revealed that the FRN effect using the LM reference method was larger than that using the REST reference method, which, in turn, was larger than that using the AVE reference method. A main effect of topographical factors was also observed [*F*_(2, 44)_ = 37.4, *p* < 0.001, partial η^2^ = 0.63], and pairwise comparisons revealed that the FRN effect was the largest at FCz, which was significantly larger than that at Cz, CPz, and Pz (ps < 0.001). The interaction between these two factors was not significant (*F* < 0.1). As clearly shown in Figure 6, the topographical pattern was similar across the LM, REST, and AVE reference approaches. The effect of the reference method on the FRN effect was mainly revealed in the amplitude differences but not in the scalp distribution.

To statistically examine the possible influence of the reference approach on the N400 effect and the magnitude of the ERP effect between the two expectancy violations, repeated-measures ANOVAs with three factors (i.e., experimental condition, reference method and ROI) were performed for the middle electrode site. A main effect of experimental condition was revealed [*F*_(1, 22)_ = 12.74, *p* = 0.002, partial η^2^ = 0.37], indicating that the N400 effect under the unrelated condition was larger than that under the related condition. Furthermore, a main effect of ROI was observed [*F*_(6, 132)_ = 13.08, *p* < 0.001, partial η^2^ = 0.37], with the maximal N400 effect occurring at Pz (mean: −3.51, *SE* = 0.32) and the minimal effect occurring at Fz (mean: −0.63, *SE* = 0.45). A main effect of reference method was also observed [*F*_(2, 44)_ = 44.29, *p* < 0.001, partial η^2^ = 0.67], indicating that the amplitude of the N400 effect observed with the LM reference approach was larger than that observed with the REST reference approach, followed by that with the AVE reference method. More importantly, the interaction between reference method and experimental condition was significant [*F*_(2, 44)_ = 6.25, *p* < 0.01, partial η^2^ = 0.22]. The *post hoc* tests and Bonferroni adjustment revealed that the N400 effect under the unrelated condition was larger than that under the related condition using all three reference methods [LM: *F*_(1, 22)_ = 11.33, *p* = 0.003, partial η^2^ = 0.34; REST: *F*_(1, 22)_ = 9.70, *p* = 0.005, partial η^2^ = 0.31; AVE: *F*_(1, 22)_ = 8.34, *p* = 0.008, partial η^2^ = 0.27], indicating that all reference approaches could capture the experimental pattern of increasing N400 effect magnitudes with increasing expectancy violations. Moreover, the *p*-value and effect sizes using the LM method were larger than those using the REST, followed by those using the AVE reference approach. Thus, all three reference methods could capture the nature of the N400 effect.

## Discussion

The current study examined whether a violation of antonym relation expectancy could induce an FRN effect and to determine the extent to which the choice of reference method influences the amplitude and scalp distribution of the experimental effect. Thus, we compared the effects of the LM, REST, and AVE reference methods on the ERP components induced by expectancy deviations of antonym relations and gambling outcomes using a within-subject design. The results revealed that the expectancy violation of antonym relations elicited N400 rather than FRN components. In addition, the reference approach influenced the magnitudes of the N400 and FRN effects but did not change the N400 and FRN responses as a function of expectancy deviation. The magnitudes of the N400 and FRN effects using the LM reference method were larger than those using the REST method, followed by those using the AVE reference approach. Moreover, although the scalp distributions of the N400 and FRN effects were similar among all three reference methods, the electrode sites that revealed significant expectancy deviation effects differed among the three reference methods.

### N400 and FRN

According to the mass univariate analysis, the statistically significant negative component in the gambling task mainly appeared at 180-330 ms and was distributed in the front-central area using all three reference methods, which reliably replicates the typical FRN effect observed in gambling tasks (Gehring and Willoughby, 2002). Thus, we reliably replicated the FRN effect. However, the negative component in the antonym task mainly appeared at 290–500 ms under both the related and unrelated conditions and had the maximum values at the more posterior electrode sites, which is consistent with the N400 component revealed in previous semantic violation tasks (Kutas and Hillyard, 1980; Kutas and Federmeier, 2000). Furthermore, the multifactor statistical analysis showed that the FRN effect was specific to the gambling task but not the antonym task, whereas the negative component at the N400 window was significant only in the antonym task but not the gambling task. These results further confirmed that the FRN effect was induced in the gambling task, but that the negative component induced in the antonym task was the N400.

N400 is a negative-going wave with a central-parietal topographic distribution that occurs at 300–600 ms and peaks at 400 ms post-stimulus onset; N400 was first observed in sematic expectancy violations in participants reading a sentence (Kutas and Hillyard, 1980). However, the temporal and spatial profiles of the N400 in the current study differed from those of Roehm et al. (2007). There were three differences between these two studies in addition to the language difference. First, the number of word pair sets was larger in our study than that in the Roehm study. The larger word sets in the current study guaranteed that each word (both X and Y) was only presented once, while word repetition of X occurred in the previous study. Second, although both studies presented the fixed sentence “*The opposite of X is Y*,” X was the first word of the fixed sentence in the Chinese sentence. The processing duration of word X was longer in this sequence, which might lead to a stable expectation about the opposite of X. Third, in our study, the ISI between the critical word Y and the previous word was 250 ms with a 50-ms per jitter step (range: 200–300 ms), while the ISI was fixed at 200 ms in the Roehm study (2007). The ISI jitter is functionally similar to high frequencies without waveform distortion (Luck, 2014). These differences might lead to the different profiles in the ERP components between these two studies. Given the observed patterns in the present study, the violation of the expected antonym relations likely induced the N400 instead of the FRN component.

Previous studies have suggested that the FRN codes violations of expectancy as feedback related to monetary loss (Gehring and Willoughby, 2002; Yu and Zhou, 2006a,b; Wu and Zhou, 2009), unexpected outcomes related to performance (Holroyd and Coles, 2002; Hajcak et al., 2007; Oliveira et al., 2007, and even stimuli of unexpected colors (Jia et al., 2007). For example, when the participants were asked to guess whether the color of the first stimulus would be the same as that of the second stimulus, the results showed that the second stimulus could induce FRN if it was an unexpected different color from the first stimulus (Jia et al., 2007). The pattern of stimulus presentation in the antonym task of our study is similar with the color guessing task in Jia et al. (2007). However, the second word in the current study induced N400 rather than FRN if it was an unexpected antonym word. Thus, an unexpected antonym word could not induce FRN, and FRN cannot codes all kinds of expectancy violations.

### Reference effect on the ERP amplitude

Generally, in the current study, all reference methods could effectively distinguish the sematic violation-evoked N400 and gambling-evoked FRN. In addition, for all experimental effects (N400 under the related and unrelated conditions and FRN), the LM reference approach elicited larger amplitudes than the REST reference approach. In addition, the amplitudes of the AVE-obtained N400 and FRN responses were even smaller than those obtained using the REST reference method, which is consistent with previous MMN (mismatch negativity) studies (Mahajan et al., 2017). The ERP effects observed in this study varied depending on the adopted reference method, which is also consistent with previous studies (Kayser et al., 2007; Yao et al., 2007; Tian and Yao, 2013; Liu et al., 2015). The LM reference method is a non-zero reference approach in which the potential of the mastoid electrode sites is contaminated by brain activity and is not zero (i.e., a positive or negative potential) (Yao, 2001). Thus, the recorded positive or negative potential at the mastoids could lead to increases or decreases in the potential measured at the active electrode sites. According to previous simulation studies, REST has been proposed as a reference-free approach and gold standard reference method (Yao, 2001, 2017; Chella et al., 2017). In the current study, we observed that the amplitudes using the LM reference approach were larger than those observed using the REST method, which might be caused by the fact that the non-zero potentials of the mastoid electrode sites obtained using the LM reference approach increased the true potentials.

The pattern in which the N400 effect was larger under the related condition than that under the unrelated condition was observed in all reference approaches. Thus, the N400 effect is a function of deviation and is not contingent on the choice of reference method. Moreover, only a slight difference was observed among the reference approaches in the extent of the significance level (*p*-value) and effect size (Cohen's d). This might be benefited from the fact that the N400 amplitude is large and the signal-to-noise ratio is also high, which led to a small influence of the reference approach on the experimental effect. The experimental effect, as measured by the amplitude differences among conditions, is an important index in ERP studies. However, the experimental effects could be influenced by the reference approach (Tian and Yao, 2013; Liang et al., 2017; She et al., 2017), which might alter the results. Hence, choosing a neutral reference approach is important. Previous simulation studies suggest that the REST method is a neutral reference method since it can approximately reconstruct an infinite reference site that is not contaminated by brain activity (Yao, 2001; Zhai and Yao, 2004; Liu et al., 2015; Chella et al., 2017). Combined with previous simulation studies, the observed results in the current study suggest that REST usually achieves more objective results, which could possibly change the significance of the ERP pattern or experimental effects compared to the other reference methods (Tian and Yao, 2013).

### Reference effect on the scalp distribution

According to the mass univariate analysis, the scalp distributions of the significant experimental effects differed among the three reference methods. In the antonym relations task, the distribution appeared similar between LM and REST, while REST showed more significant sites in the right hemisphere, which was consistent with the typical distribution of N400 effects (Luck, 2014). The distribution of the FRN effect also appeared similar between LM and REST, while the significant electrode sites were mainly focused in the front-central area using the REST reference approach, which is consistent with the typical distribution of FRN effects (Gehring and Willoughby, 2002). In addition, the AVE-obtained N400 effect and FRN effect were reversed in polarity (positive amplitude) at certain electrode sites, which is consistent with previous studies (Luck, 2014). The reverse pattern observed using the AVE reference method is common since the potentials of all active sites are subtracted from the sum of the voltages across all electrode sites, which is zero at each time point. This leads to positive effects along with negative effects. Thus, researchers should be cautious when using the AVE reference approach.

To statically assess the scalp distribution of N400 and FRN, repeated-measured ANOVA of the amplitude of N400 and FRN at the middle electrode site was conducted. Both the topographical distribution and statistical analysis revealed similar patterns, and only a few differences were observed among all reference approaches. This finding is consistent with previous studies (Tian and Yao, 2013; Liang et al., 2017; Yang et al., 2017). Given the results of these two analyses, the scalp distribution of the experimental effect obtained by using the REST method was more consistent with the results of previous studies, highlighting the objectivity of the REST reference approach.

## Conclusion

The results of the current study indicate that expectancy violations in the antonym relations context induced an N400 effect instead of an FRN effect. Considering the theoretical and simulated evidence, we suggest that REST is an optional reference method to be used in future ERP data analyses. These findings contribute to other empirical investigations regarding the choice of reference approach in ERP domains.

## Ethics statement

This study was carried out in accordance with the recommendations of the Ethics Committee of the School of Psychological and Cognitive Sciences, Peking University with written informed consent from all subjects. All subjects gave written informed consent in accordance with the Declaration of Helsinki. The protocol was approved by the Ethics Committee of the School of Psychological and Cognitive Sciences, Peking University.

## Author contributions

YL, YcW, and XZ designed the study. YL conducted the experiment. YL, YcW, and BZ analyzed and interpreted the data. YL, YhW, and XZ wrote the paper. All authors contributed to and have approved the final version of the manuscript.

### Conflict of interest statement

The authors declare that the research was conducted in the absence of any commercial or financial relationships that could be construed as a potential conflict of interest.
